# Integrated Pest Management of Fruit Mites in Apple Orchards: Biological and Chemical Control, Resistance Management, and Regional Perspectives from Southeastern Kazakhstan

**DOI:** 10.3390/insects17080795

**Published:** 2026-07-31

**Authors:** Assel A. Karabayeva, Bakyt K. Kopzhasarov, Gulnar K. Ziyaeva, Nazira M. Duzbayeva, Rabiga M. Kudaibergenova

**Affiliations:** 1 Department of Biology, Faculty of Natural Sciences, M.Kh. Dulaty Taraz University, Taraz 080000, Kazakhstan; aa.karabaeva@dulaty.kz (A.A.K.); gk.ziyaeva@dulaty.kz (G.K.Z.); 2 LLP “Zh. Zhiembayev Kazakh Research Institute for Plant Protection and Quarantine”, Kultobe 1, Rakhat Microdistrict, Nauryzbai District, Almaty 050000, Kazakhstan; plantprotectionkz@gmail.com; 3 Department of Chemistry and Chemical Technology, Faculty of Technology, M.Kh. Dulaty Taraz University, Taraz 080000, Kazakhstan

**Keywords:** fruit mites, apple orchards, integrated pest management, biological control, acaricide resistance, predatory mites, sustainable agriculture, southeastern Kazakhstan

## Abstract

Fruit mites, particularly *Panonychus ulmi*, *Tetranychus urticae*, and eriophyid mites, are among the most economically important arthropod pests in apple orchards, causing significant reductions in photosynthetic efficiency, fruit quality, and yield. Their management has traditionally relied on chemical acaricides; however, the widespread development of resistance, adverse effects on beneficial arthropods, and increasing environmental concerns have highlighted the limitations of pesticide-dependent control strategies. This review critically evaluates current advances in the management of major fruit mite species affecting apple orchards, with particular emphasis on *Panonychus ulmi*, *Tetranychus urticae*, and eriophyid mites. The biology, ecology, and economic significance of these pests are examined together with contemporary chemical control strategies, mechanisms of acaricide resistance, and sustainable resistance management. Particular attention is given to predatory mites, conservation biological control, habitat management, and ecological engineering as essential components of Integrated Pest Management (IPM). The review also examines the integration of biological, chemical, and cultural control measures within IPM programs and provides a regional perspective on fruit mite management in Southeastern Kazakhstan, highlighting local challenges, knowledge gaps, and opportunities for implementing ecologically based IPM systems. Emerging approaches, including precision agriculture, digital monitoring, molecular diagnostics, and climate-adaptive pest management, are also assessed. The available evidence indicates that sustainable fruit mite management requires a transition from pesticide-centered control toward integrated, ecologically resilient orchard protection systems in which biological control and IPM serve as the foundation, while chemical control functions as a targeted supporting tool. To our knowledge, no previous review has comprehensively integrated biological control, acaricide resistance management, ecologically based IPM, and region-specific challenges for fruit mite management in Southeastern Kazakhstan into a unified framework while also identifying key research priorities for Central Asia, including resistance surveillance, biodiversity conservation, climate adaptation, and digital decision-support technologies.

## Simple Summary

Fruit mites are among the most damaging pests of apple orchards because they reduce leaf function, fruit quality, and crop yield. Farmers have traditionally relied on chemical pesticides to control these pests, but repeated use has led to pesticide resistance, environmental concerns, and negative effects on beneficial organisms. This review explains how sustainable management can be achieved by combining biological, chemical, and cultural control methods within Integrated Pest Management, an approach that reduces pesticide use while maintaining effective pest control. Particular attention is given to natural enemies, resistance management, and environmentally friendly practices that improve long-term orchard health. The review also highlights current challenges and research needs in Southeastern Kazakhstan, one of the major apple-growing regions and the center of origin of the wild apple (*Malus sieversii*). In addition, new technologies such as digital monitoring and precision agriculture are discussed as promising tools for future pest management. This review provides practical information that may help researchers, agricultural specialists, and apple growers develop more sustainable and environmentally responsible strategies for managing fruit mites.

## 1. Introduction

Sustainable apple production increasingly depends on effective management of arthropod pests while minimizing pesticide dependence and environmental impacts. The increasing demand for high-quality apples has intensified orchard production systems, leading to greater reliance on plant protection measures to minimize yield losses caused by pests and diseases [1,2]. However, intensive pest management practices have also raised concerns regarding pesticide overuse, environmental contamination, biodiversity loss, and the development of resistance in target pest populations [3,4,5].

Among the numerous arthropod pests affecting apple orchards, fruit mites are considered one of the most destructive and economically significant groups. Species such as the European red mite (*Panonychus ulmi* Koch) and the two-spotted spider mite (*Tetranychus urticae* Koch) cause severe damage through leaf feeding, resulting in chlorosis, reduced photosynthetic activity, premature defoliation, impaired fruit development, and decreased orchard productivity [6,7]. The economic impact of mite infestations is further exacerbated by their rapid reproductive capacity, short generation times, and strong ability to develop resistance to acaricides [8].

For several decades, management of fruit mites has relied predominantly on chemical control. Although synthetic acaricides have provided effective short-term suppression, intensive and repeated applications have resulted in widespread resistance development, disruption of natural enemy populations, pesticide residues in agricultural products, and increasing ecological risks [5,9,10]. Consequently, there is growing international interest in developing sustainable pest management strategies that reduce dependence on conventional pesticides while maintaining effective control of economically important orchard pests.

Biological control has emerged as a cornerstone of sustainable fruit production systems. Predatory mites, particularly species belonging to the family Phytoseiidae, play a crucial role in regulating phytophagous mite populations in commercial orchards [11,12]. In addition, habitat management, conservation biological control, ecological engineering, and selective pesticide use have been increasingly incorporated into Integrated Pest Management (IPM) programs aimed at enhancing ecosystem services and improving long-term orchard resilience [13,14,15]. Recent studies have demonstrated that diversified agricultural landscapes and ecologically based management practices can significantly strengthen natural pest regulation and contribute to sustainable crop production [16,17].

Despite substantial advances in biological control and IPM, important challenges remain. Resistance evolution in mite populations, climate-driven shifts in pest dynamics, limited adoption of ecological management approaches, and the need for region-specific IPM programs continue to constrain sustainable fruit mite management [8,18]. Furthermore, while numerous reviews have examined general pest management or biological control in orchard systems, a comprehensive synthesis focusing specifically on fruit mites and integrating biological control, resistance management, and modern IPM strategies remains limited.

Particularly important is the need to evaluate sustainable fruit mite management under the environmental conditions of Central Asia, where apple production represents a strategically important agricultural sector. Southeastern Kazakhstan constitutes one of the principal apple-growing regions of the country and, as the center of origin of *Malus sieversii*, represents a unique agroecological region characterized by high apple biodiversity and diverse orchard systems. These characteristics provide an important setting for developing and evaluating sustainable Integrated Pest Management (IPM) strategies under locally relevant ecological conditions. Developing ecologically sound protection systems for fruit mite control is therefore essential for improving the sustainability and competitiveness of regional apple production.

The aim of this review is to critically evaluate current advances in the biological control and integrated pest management of fruit mites in apple orchards. To the best of our knowledge, this is one of the first reviews to integrate biological control, acaricide resistance management, ecologically based IPM strategies, precision agriculture, and regional evidence from Southeastern Kazakhstan into a unified framework for sustainable fruit mite management. By combining these complementary aspects, this review identifies current challenges, research gaps, and future opportunities for developing sustainable and region-specific orchard protection systems in Central Asia.

## 2. Literature Search Methodology

A literature search was conducted using the Scopus, Web of Science, PubMed, Google Scholar, ScienceDirect, MDPI, SpringerLink, and Wiley databases. The search strategy combined keywords related to fruit mites, biological control, acaricide resistance, and Integrated Pest Management (IPM) using Boolean operators. An example of the search string was: (“*Panonychus ulmi*” OR “*Tetranychus urticae*” OR “fruit mites” OR eriophyid mites) AND (“apple orchards” OR apple) AND (“biological control” OR “integrated pest management” OR IPM OR “acaricide resistance”). Additional searches were performed using the terms “predatory mites”, “habitat management”, and “sustainable orchard protection”.

Priority was given to peer-reviewed publications published between 2010 and 2026, while seminal earlier studies were included when relevant. A total of 312 publications were initially identified, of which 97 were selected for detailed analysis. The selected publications covered the following major topics: mite biology (20), acaricide resistance (17), biological control (24), habitat management (13), and IPM implementation (23). Regional publications from Kazakhstan and Central Asia were additionally reviewed to identify local challenges, knowledge gaps, and region-specific management perspectives.

Publications were included if they focused on fruit mites in apple orchards, biological control, acaricide resistance, Integrated Pest Management (IPM), or sustainable orchard protection. Conference abstracts, duplicate records, non-peer-reviewed publications, studies lacking sufficient scientific information, and publications unrelated to apple production systems or fruit mite management were excluded.

## 3. Biology, Ecology and Economic Importance of Fruit Mites in Apple Orchards

Fruit mites are among the most persistent arthropod pests affecting apple production worldwide. Their economic significance is primarily associated with rapid population growth, short generation times, high reproductive potential, and their ability to thrive in intensive orchard systems characterized by high planting density and frequent pesticide applications. Unlike many insect pests, phytophagous mites often remain unnoticed during the early stages of infestation due to their small size, allowing populations to increase rapidly before visible symptoms become apparent [6,19].

Among the numerous mite species reported in apple orchards, only a limited number consistently cause economically significant damage. The European red mite (*Panonychus ulmi* Koch), the two-spotted spider mite (*Tetranychus urticae* Koch), and the apple rust mite (*Aculus schlechtendali* Nalepa) are regarded as the most important species causing direct economic damage in commercial apple production systems [6,9,20]. Their distribution, biology, feeding behavior, and economic impact differ considerably, requiring species-specific management approaches.

### 3.1. Major Fruit Mite Species Affecting Apple Production

The European red mite (*P. ulmi*) remains one of the most destructive pests of apple orchards in temperate regions. This species overwinters as eggs deposited on bark and branches, enabling rapid population establishment during spring. Feeding activity reduces chlorophyll content and photosynthetic efficiency, ultimately affecting fruit size, coloration, and yield [21]. Due to its multiple generations per season and strong capacity for resistance development, *P. ulmi* continues to be a major challenge for orchard managers despite decades of control efforts [22,23].

The two-spotted spider mite (*T. urticae*) is characterized by an exceptionally broad host range and high ecological adaptability. Unlike *P. ulmi*, outbreaks of *T. urticae* are frequently associated with hot and dry environmental conditions and are often intensified following repeated applications of broad-spectrum pesticides that disrupt natural enemy communities [24]. Consequently, the species is increasingly regarded as an indicator of ecological imbalance within orchard ecosystems.

Apple rust mite (*A. schlechtendali*) has historically been considered a secondary pest. However, recent studies suggest that its role within orchard ecosystems is more complex than previously assumed. Moderate populations may serve as an alternative food source for predatory mites, thereby contributing to biological control stability. However, excessive population growth can result in leaf bronzing, reduced chlorophyll content and photosynthetic activity, ultimately leading to reduced fruit quality and productivity [21]. This dual ecological role highlights the importance of threshold-based management rather than routine suppression. The major fruit mite species affecting apple orchards and their direct economic significance are summarized in Table 1.

### 3.2. Population Dynamics and Ecological Drivers

Fruit mite population dynamics are strongly influenced by temperature, humidity, host plant condition, and orchard management practices. Climatic warming has emerged as one of the most important drivers of increasing mite pressure in many fruit-growing regions [25]. Elevated temperatures are particularly important for tetranychid mites such as *Panonychus ulmi* and *Tetranychus urticae*, as they substantially shorten developmental time and increase the number of generations completed during the growing season. *T. urticae*, in particular, exhibits rapid population growth under warm and dry conditions due to its high reproductive potential and short life cycle. Consequently, rising temperatures may disproportionately increase the risk of mite outbreaks compared with many other orchard pests.

However, climate alone does not fully explain fruit mite population dynamics. Orchard management practices, including pesticide use, habitat management, irrigation practices, and the conservation of natural enemies, also play a critical role in shaping mite populations in apple orchards [9]. Intensive pesticide programs may eliminate predatory mites and other beneficial arthropods, creating conditions favorable for secondary pest outbreaks [15]. This phenomenon has been repeatedly documented for both *P. ulmi* and *T. urticae*, where suppression of natural enemies results in rapid pest resurgence despite continued pesticide applications [9,22].

These observations challenge the traditional perception that fruit mite outbreaks are primarily a consequence of favorable weather conditions. Instead, increasing evidence suggests that many outbreaks represent ecological responses to disruptions within orchard food webs.

In addition to temperature and humidity, other environmental factors can significantly influence fruit mite population dynamics. Dust accumulation on foliage may promote mite outbreaks by reducing the effectiveness of natural enemies and altering leaf surface characteristics. Plant water status is also an important ecological driver, as drought stress and insufficient irrigation can increase host plant susceptibility and create favorable conditions for mite development and reproduction. These factors may be particularly relevant in semi-arid apple-growing regions, including southeastern Kazakhstan, where high summer temperatures, dust exposure, and periodic water deficits frequently occur.

### 3.3. Damage Mechanisms and Economic Impact

Fruit mites damage apple trees primarily through piercing-sucking feeding activity. Feeding causes destruction of mesophyll cells, reductions in chlorophyll concentration, impairment of photosynthetic processes, and alterations in plant physiological functions [21]. As infestation levels increase, cumulative feeding injury results in reduced carbohydrate accumulation, premature leaf senescence, diminished fruit quality, and lower marketable yields.

Economic losses associated with fruit mites are often underestimated because damage develops gradually and may not become visible until populations have exceeded economic thresholds. Moreover, indirect economic losses associated with increased pesticide applications, resistance management, disruption of biological control programs, and additional monitoring and phytosanitary measures can substantially increase the overall economic impact of fruit mite infestations [26].

A critical issue in modern apple production is that economic damage is no longer determined solely by pest density. Increasingly, economic outcomes depend on the stability of orchard ecosystems and the effectiveness of integrated management programs. Consequently, the economic significance of fruit mites should be evaluated not only in terms of direct crop losses but also in relation to their influence on long-term orchard sustainability.

### 3.4. Implications for Sustainable Pest Management

The ecological characteristics of fruit mites indicate that sustainable management cannot rely on chemical control alone. Instead, long-term suppression requires the integration of ecological monitoring, conservation of predatory mites, resistance management, and orchard-specific cultural practices. These principles provide the conceptual framework for the following sections, which discuss biological control agents, acaricide resistance, and integrated pest management strategies in greater detail.

## 4. Chemical Control and Resistance Development

Chemical control has historically been the cornerstone of fruit mite management in commercial apple orchards. Since the mid-twentieth century, acaricides have provided rapid and economically effective suppression of phytophagous mite populations, contributing substantially to yield protection and fruit quality maintenance [27,28].

Despite these benefits, the long-term sustainability of acaricide-dependent management has become increasingly questioned. Continuous reliance on chemical interventions has generated significant ecological and economic challenges, including resistance development, disruption of natural enemy populations, secondary pest outbreaks, and increasing regulatory restrictions on pesticide use [23,29].

### 4.1. Conventional Acaricide-Based Management

Modern fruit mite management relies on several acaricide groups with distinct modes of action, including mitochondrial electron transport inhibitors (METIs), lipid synthesis inhibitors, growth regulators, and neurotoxic compounds [30]. These acaricides remain important components of commercial fruit mite management because they provide rapid suppression of phytophagous mite populations. However, repeated use of the same active ingredients or compounds sharing similar modes of action has accelerated selection pressure on mite populations. Unlike many insect pests, phytophagous mites possess biological characteristics that facilitate rapid adaptation, including short generation times, high fecundity, arrhenotokous reproduction, and substantial genetic variability [23,31]. The major acaricide groups used in Integrated Pest Management (IPM) of fruit mites in apple orchards, together with their representative active ingredients, modes of action, target stages, major limitations, and resistance risk, are summarized in Table 2.

Although these compounds continue to play an important role in orchard protection, their effectiveness increasingly depends on proper rotation strategies and integration with non-chemical management approaches [32].

### 4.2. Resistance Development in Fruit Mite Populations

Resistance evolution is currently regarded as one of the greatest threats to sustainable fruit mite management worldwide. Among agricultural arthropods, spider mites are frequently cited as some of the most resistance-prone pest species due to their short generation times, high reproductive potential, and remarkable adaptive capacity [23,33]. Acaricide resistance has been documented in association with nearly all major acaricide groups used against phytophagous mites. This widespread occurrence indicates that sustainable fruit mite management cannot rely solely on the introduction of new active ingredients. Instead, effective resistance management requires the integration of biological control, rotation of acaricides with different modes of action, resistance monitoring, and ecologically based IPM strategies. The current status of documented resistance in major fruit mite species is summarized in Table 3.

As shown in Table 3, documented acaricide resistance is well established in *Panonychus ulmi* and *Tetranychus urticae*, whereas comparable information for eriophyid mites remains very limited. This apparent difference may partly reflect the limited number of studies rather than a true absence of resistance. Nevertheless, increasing reliance on repeated acaricide applications and the limited availability of registered products may increase future resistance risks in eriophyid mite populations. Further research is therefore needed to strengthen resistance monitoring, elucidate resistance mechanisms, and develop sustainable management strategies for eriophyid mite species.

Resistance mechanisms include target-site mutations, enhanced metabolic detoxification, reduced penetration, and behavioral adaptations that decrease exposure to toxic compounds [23,34]. The challenge is further intensified by the extraordinary adaptive capacity of spider mites. Studies indicate that resistance can evolve within relatively short periods when strong selection pressure is maintained, particularly in intensive orchard systems where multiple treatments are applied annually [33]. Consequently, the introduction of new active ingredients often provides only temporary solutions before resistance emerges.

Importantly, acaricide resistance should not be viewed solely as a genetic phenomenon. Increasing evidence suggests that resistance development is fundamentally linked to management decisions. Frequent acaricide applications, insufficient mode-of-action rotation, and preventive rather than threshold-based treatments significantly accelerate resistance evolution [22,35].

### 4.3. Ecological Consequences of Chemical Control

Many acaricides exhibit varying degrees of toxicity toward beneficial arthropods, particularly predatory mites belonging to the family Phytoseiidae [22,24]. Disruption of these natural enemies may reduce biological control efficiency and contribute to pest resurgence following pesticide applications [22,24]. In addition, intensive acaricide use may reduce arthropod biodiversity, increase pesticide residues, and contribute to increasingly restrictive pesticide regulations aimed at reducing environmental risks and protecting non-target organisms [4,36].

## 5. Sustainable Resistance Management

Sustainable resistance management is an essential component of modern Integrated Pest Management (IPM) programs. Historically, resistance management was often viewed as a pesticide-centered issue focused primarily on replacing ineffective products with new active ingredients. However, growing evidence indicates that resistance development is fundamentally an ecological and evolutionary process influenced by management practices, orchard biodiversity, and pest population dynamics [29,35]. Therefore, long-term resistance mitigation requires a systems-based approach rather than reliance on chemical innovation alone.

### 5.1. Mechanisms and Drivers of Resistance Evolution

Resistance in fruit mite populations arises through a combination of genetic variability and selection pressure imposed by repeated pesticide applications [23]. Individuals possessing resistance-conferring traits survive treatment and subsequently contribute disproportionately to future generations.

Several resistance mechanisms have been identified in *Panonychus ulmi* and *Tetranychus urticae*, including target-site mutations, enhanced metabolic detoxification, reduced pesticide penetration, and behavioral avoidance responses [23,34]. Among these mechanisms, metabolic resistance mediated by detoxification enzymes such as cytochrome P450 monooxygenases, esterases, and glutathione S-transferases is particularly important because it can confer cross-resistance to multiple acaricide classes [35].

A critical challenge is that resistance development is rarely driven by a single factor. Intensive pesticide use, limited mode-of-action diversity, frequent preventive treatments, and inadequate monitoring collectively increase selection pressure and accelerate resistance evolution [29]. Consequently, resistance should be regarded as a management outcome rather than merely a biological characteristic of pest populations.

### 5.2. Resistance Monitoring and Early Detection

Traditional resistance monitoring relies primarily on laboratory bioassays that compare the responses of field populations with those of susceptible reference strains [37,38]. Although these methods remain valuable, they are labor-intensive and may fail to detect resistance during its early stages. Recent advances in molecular diagnostics have substantially improved resistance monitoring by enabling the identification of resistance-associated mutations and metabolic markers, thereby facilitating earlier assessment of resistance risk and supporting more precise resistance management [39,40]. Nevertheless, despite these technological advances, resistance monitoring remains underutilized in many commercial apple orchards. In practice, resistance is often recognized only after noticeable declines in field efficacy have occurred, by which time resistant populations may already be widespread [38,39]. This gap between scientific advances and their practical implementation remains one of the major challenges of sustainable resistance management.

### 5.3. Reducing Selection Pressure Through IPM

Modern resistance management increasingly focuses on reducing selection pressure rather than solely responding to resistance after it emerges [32].

Economic threshold-based decision-making is particularly important because it prevents routine pesticide applications when pest populations remain below damaging levels [14]. Such practices reduce both pesticide use and resistance selection pressure.

Furthermore, biological control contributes directly to resistance management by lowering pest populations independently of chemical interventions [41]. Predator-mediated suppression reduces reliance on acaricides and may slow the spread of resistant genotypes within mite populations.

These findings suggest that biological control should not be viewed solely as a pest suppression tool but also as an indirect mechanism for resistance mitigation.

### 5.4. Acaricide Rotation and Mode-of-Action Diversity

Rotation of acaricides with different modes of action remains one of the most widely recommended strategies for resistance management [32]. By alternating compounds that target different physiological pathways, the probability that resistant individuals survive successive treatments is reduced. Consequently, the IRAC mode-of-action classification system has become a fundamental tool for planning resistance management programs [30]. However, practical implementation is often constrained by limited product availability, economic considerations, and regional registration restrictions, which may reduce opportunities for effective rotation [42]. Moreover, mode-of-action rotation alone cannot fully prevent resistance development, as resistance has evolved even in production systems implementing rotational programs when overall pesticide dependence remained high [29,33]. Therefore, acaricide rotation should be regarded as a necessary, but not sufficient, component of sustainable resistance management.

### 5.5. Future Directions for Sustainable Resistance Management

Future resistance management programs are expected to increasingly integrate ecological, technological, and molecular approaches. Promising directions include molecular resistance diagnostics, predictive resistance modeling, precision pesticide application technologies, enhanced biological control programs, biodiversity-based pest suppression, and ecological engineering of orchard systems [40,43]. In addition, climate change is expected to further complicate resistance management by accelerating pest development, increasing the frequency of pest outbreaks, and potentially intensifying selection pressure for resistance [25]. Consequently, adaptive management strategies capable of responding to changing environmental conditions will become increasingly important for sustaining the long-term effectiveness of resistance management programs.

Table 4 summarizes the principal components of sustainable fruit mite management within IPM programs, highlighting their primary roles, advantages, limitations, and contributions to long-term sustainability.

## 6. Biological Control of Fruit Mites

Biological control represents one of the most promising components of sustainable fruit mite management in modern apple production systems. Unlike conventional chemical control, biological control aims to regulate pest populations through naturally occurring ecological interactions while minimizing environmental impacts and preserving biodiversity [11,12]. The increasing adoption of Integrated Pest Management (IPM) programs worldwide has further strengthened the role of biological control as a cornerstone of sustainable orchard protection.

### 6.1. Natural Enemies of Fruit Mites in Apple Orchards

Natural enemies constitute the foundation of biological control programs targeting phytophagous mites in apple orchards. Among them, predatory mites of the family Phytoseiidae are regarded as the most effective and economically important biological control agents because of their close ecological association with spider mites and their compatibility with integrated pest management (IPM) programs [44,45].

Species such as *Typhlodromus pyri*, *Amblyseius andersoni*, *Neoseiulus fallacis*, and *Phytoseiulus persimilis* have demonstrated high efficacy in suppressing populations of *Panonychus ulmi*, *Tetranychus urticae*, and other economically important phytophagous mites under commercial orchard conditions and experimental studies [46]. Among these species, *T. pyri* is considered one of the most successful biological control agents in temperate apple-growing regions because of its broad prey range, tolerance to variable environmental conditions, and compatibility with selective pesticide programs [47]. Furthermore, phytoseiid mites can exploit alternative food resources, particularly pollen, enabling predator populations to persist even when phytophagous mite densities remain low [48].

Although phytoseiid mites are the principal natural enemies of spider mites, other predators also contribute to biological control. Predatory ladybird beetles of the genus *Stethorus* (Coleoptera: Coccinellidae), particularly *Stethorus punctillum*, are highly specialized predators of tetranychid mites and can effectively suppress spider mite outbreaks when prey populations reach high densities [49,50]. The effectiveness of biological control depends not only on predator abundance but also on climatic conditions, prey availability, habitat complexity, pesticide selectivity, and interactions among natural enemy species [51]. Consequently, successful biological control in apple orchards relies on the conservation of diverse natural enemy communities rather than on a single predator species or release programs alone. Long-term ecological stability and the preservation of functional biodiversity are therefore considered essential components of sustainable fruit mite management.

### 6.2. Conservation Biological Control

Conservation biological control aims to enhance naturally occurring enemy populations through habitat management and ecological diversification rather than through repeated introductions of biological agents [12,44,47,52].

Increasing evidence suggests that diversified orchard ecosystems support more stable predator communities and improve natural pest suppression [11,16]. Floral resources, hedgerows, cover crops, and other non-crop vegetation provide shelter, alternative prey, pollen, nectar, and overwintering habitats for beneficial arthropods, thereby enhancing conservation biological control [12,53].

Particularly important is the maintenance of phytoseiid predator populations during periods of low pest abundance. In many orchards, predator populations may decline before phytophagous mite populations increase, creating a temporal mismatch that can reduce the effectiveness of biological control [54,55]. Habitat management strategies can reduce this mismatch by supporting predator populations throughout the growing season.

### 6.3. Biological Control Using Predatory Mite Releases

Augmentative biological control involves the deliberate release of commercially produced predatory mites to suppress pest populations [12]. Species such as *Phytoseiulus persimilis* are widely used in greenhouse production systems, whereas *Amblyseius andersoni* has become an important biological control agent in apple orchards because of its compatibility with integrated pest management programs and its persistence under field conditions [47,56].

Field studies have demonstrated that predator releases can significantly reduce populations of *T. urticae* and *P. ulmi* under favorable environmental conditions [57]. However, the effectiveness of predator releases varies considerably among production systems owing to differences in environmental conditions, orchard management practices, and pesticide regimes [47,51].

One of the major limitations of augmentative biological control is the difficulty of establishing persistent predator populations under open-field orchard conditions. Climatic variability, dispersal losses, pesticide exposure, and limited prey availability frequently reduce predator establishment and long-term persistence [47,58]. As a consequence, repeated releases are often required, increasing implementation costs and reducing their economic attractiveness for growers. These limitations have contributed to the growing emphasis on conservation biological control, which seeks to preserve naturally occurring predator populations through habitat management and selective pesticide use rather than relying on repeated augmentative releases.

### 6.4. Emerging Biological Control Approaches

Recent research has expanded beyond traditional predator–prey interactions and increasingly focuses on microbial-based control strategies and other emerging approaches that complement existing IPM programs [59].

Entomopathogenic fungi, including *Beauveria bassiana*, *Metarhizium anisopliae*, and *Cordyceps fumosorosea*, have demonstrated efficacy against several phytophagous mite species under both laboratory and field conditions [60]. Although fungal biocontrol agents offer environmentally friendly alternatives to conventional pesticides, their performance remains highly dependent on humidity and temperature conditions.

Similarly, semiochemical-based approaches, including herbivore-induced plant volatiles (HIPVs) and predator-attracting chemical cues, are emerging as innovative tools for enhancing biological control efficiency [61,62]. These technologies aim to strengthen natural enemy populations without direct pest suppression interventions.

Despite promising developments, most emerging biological control technologies remain at relatively early stages of commercial adoption. Additional research is required to determine their long-term effectiveness under diverse orchard conditions.

### 6.5. Critical Challenges and Future Opportunities

Although biological control is widely recognized as a key component of sustainable mite management, its implementation in commercial orchards remains challenging. Compared with chemical control, biological control generally provides slower pest suppression, requires detailed ecological knowledge, and its effectiveness may vary with climatic conditions, orchard management practices, and seasonal dynamics [47,51].

Furthermore, many studies continue to evaluate biological control success primarily in terms of pest suppression. Such assessments may underestimate the broader ecosystem services provided by biological control, including biodiversity conservation, reduced selection for pesticide resistance, and improved long-term resilience of orchard ecosystems [16,63].

A common misconception in orchard pest management is that biological control should completely replace chemical control. However, contemporary evidence supports an integrated approach in which biological control complements rather than replaces chemical interventions. Within modern IPM programs, biological control is regarded as the ecological foundation that enhances long-term pest regulation while reducing reliance on chemical inputs [16,63].

Therefore, future research should focus not only on identifying new biological control agents but also on optimizing ecological conditions that enhance the effectiveness of existing natural enemy communities. Such an approach is likely to provide more durable and economically viable solutions for fruit mite management than continual reliance on novel chemical inputs.

## 7. Integrated Pest Management Strategies in Apple Orchards

Integrated Pest Management (IPM) has emerged as the most widely accepted framework for sustainable management of fruit mites in modern apple production systems. Unlike conventional pest control approaches that rely primarily on pesticide applications, IPM integrates biological, chemical, cultural, and ecological management practices to maintain pest populations below economically damaging levels while minimizing environmental impacts and preserving ecosystem services [10,14,64].

The growing importance of IPM in apple orchards is driven by several interconnected challenges, including increasing acaricide resistance, stricter pesticide regulations, biodiversity conservation requirements, and the need to improve long-term sustainability of fruit production systems [9,15,24]. Consequently, IPM is no longer viewed merely as a collection of pest control techniques but rather as a decision-making framework that integrates ecological knowledge into orchard management.

### 7.1. Monitoring and Economic Thresholds

Effective monitoring represents the foundation of successful integrated pest management (IPM) programs because fruit mite populations can increase rapidly under favorable environmental conditions, making early detection essential for preventing economically significant outbreaks [65]. Monitoring programs typically involve the regular assessment of mite abundance on leaves, shoots, and fruiting structures throughout the growing season, and the resulting data are compared with established economic thresholds to determine whether management intervention is justified [66].

The economic threshold concept is one of the most important distinctions between IPM and conventional pest management. Under traditional pesticide-based systems, treatments are frequently applied according to predetermined schedules regardless of actual pest density. In contrast, IPM interventions are implemented only when monitoring data indicate that economic damage is likely to occur [14].

Despite widespread acceptance of threshold-based decision-making, considerable variation exists among regions and production systems. Most currently recommended economic thresholds are based on studies conducted in Europe and North America, and threshold values developed under one set of climatic and agronomic conditions may not be directly transferable to other regions [67]. This limitation is particularly relevant for emerging apple-growing regions, including Central Asia, where these thresholds have not yet been comprehensively validated under local agroecological conditions and region-specific economic thresholds remain scarce.

### 7.2. Integration of Biological and Chemical Control

Historically, biological control and pesticide applications were frequently viewed as mutually exclusive approaches. However, extensive field experience has demonstrated that successful mite management often depends on carefully balancing these components within a unified management system [64].

Selective acaricides with reduced toxicity toward predatory mites have improved the compatibility between chemical and biological control measures [22]. When applied according to monitoring-based recommendations and resistance management principles, these products can effectively suppress pest outbreaks while preserving beneficial arthropod populations [68]. Nevertheless, complete compatibility remains difficult to achieve because even selective acaricides may adversely affect predator reproduction, dispersal, and long-term population persistence [69]. Therefore, pesticide selectivity should be evaluated not only in terms of short-term mortality but also with respect to its broader ecological effects on natural enemy communities.

Increasing evidence suggests that orchard systems maintaining stable predator populations often require fewer chemical interventions over time [12,46]. This observation supports the concept that biological control should serve as the ecological foundation of IPM programs, whereas chemical control should function primarily as a corrective tool when pest populations exceed economic thresholds.

### 7.3. Cultural and Ecological Management Practices

Cultural control strategies play an essential role in reducing pest establishment and enhancing orchard resilience. Unlike direct control measures, cultural practices influence the ecological conditions that determine pest population development [52].

Several orchard management practices have been associated with improved suppression of fruit mites, including pruning to improve canopy aeration, optimization of irrigation regimes, balanced fertilization, and the removal of heavily infested plant material [12,52]. These cultural practices create less favorable conditions for phytophagous mites and contribute to more stable pest regulation while complementing monitoring, biological control, and selective chemical interventions within integrated pest management programs.

However, ecological diversification is not universally beneficial. Excessive vegetation complexity may occasionally increase competition among natural enemies or provide alternative hosts for pest species [37]. Therefore, ecological engineering strategies must be adapted to local agroecological conditions rather than applied as universal solutions.

A common weakness of many IPM programs is the tendency to emphasize direct pest suppression while underestimating the importance of ecological processes that regulate pest populations naturally. Sustainable orchard management requires a shift from reactive pest control toward proactive ecosystem management.

### 7.4. Resistance-Aware IPM Programs

Effective resistance management within IPM programs relies on the rotation of acaricides with different modes of action, reduction in unnecessary pesticide applications, monitoring of resistance development, conservation of biological control agents, application of economic thresholds, and integration of non-chemical control measures [32]. These strategies represent fundamental components of integrated pest management and collectively reduce selection pressure while maintaining the long-term effectiveness of acaricides.

A critical misconception is that resistance can be solved simply through the introduction of new active ingredients. Historical experience repeatedly demonstrates that most newly introduced pesticides eventually face resistance challenges when used intensively [29,33]. Therefore, sustainable resistance management depends more on reducing selection pressure than on continuously replacing older products.

### 7.5. Toward Ecologically Based IPM Systems

**Figure 1:**
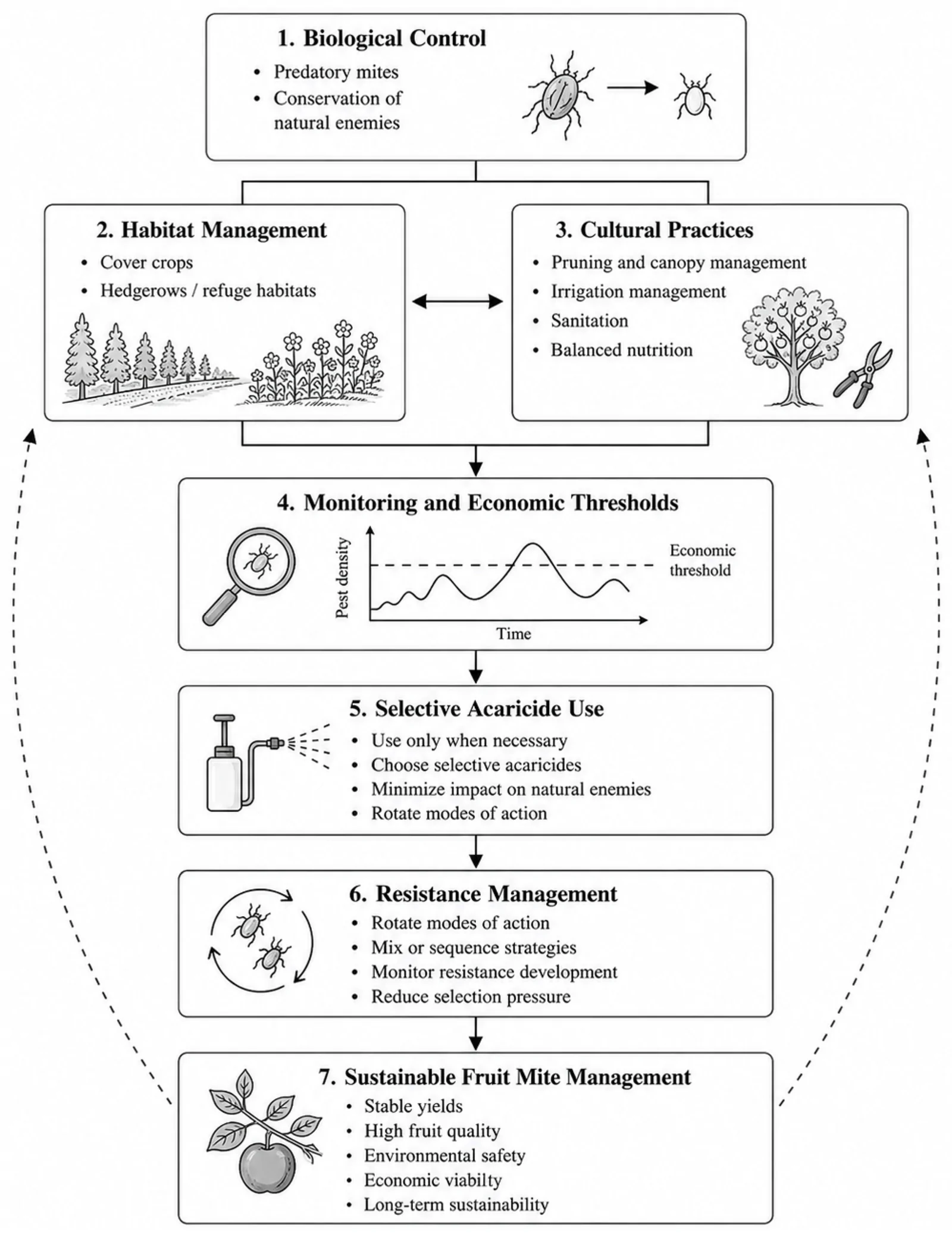
Conceptual framework of integrated pest management (IPM) for sustainable fruit mite management in apple orchards.

Contemporary IPM programs increasingly emphasize ecologically based management approaches that utilize natural ecosystem processes to regulate pest populations [16,17]. These approaches seek to enhance biodiversity, strengthen biological control, improve landscape connectivity, and reduce dependence on chemical inputs. Rather than aiming for complete pest eradication, ecologically based IPM seeks to maintain pest populations below established economic thresholds while promoting long-term ecosystem stability and resilience [11]. Recent large-scale studies indicate that diversified agricultural systems frequently achieve more stable pest regulation and greater long-term sustainability than highly simplified production systems [16,17]. Nevertheless, successful implementation remains constrained by economic considerations, regional variability, and gaps in ecological knowledge. Consequently, modern IPM increasingly relies on systems-based approaches that integrate biological control, habitat management, cultural practices, monitoring, selective acaricide use, and resistance management within a unified ecological framework. Figure 1 illustrates the interactions among these complementary components in sustainable fruit mite management in apple orchards.

## 8. Fruit Mite Management in Southeastern Kazakhstan

Southeastern Kazakhstan represents one of the most important apple-producing regions in Central Asia and is widely recognized as the historical center of apple diversity associated with *Malus sieversii*, the wild progenitor of cultivated apple varieties [70,71,72]. The region includes the Almaty, Zhetysu, and adjacent foothill zones characterized by favorable climatic conditions for apple cultivation [70]. However, these same environmental conditions also create suitable habitats for the development of numerous arthropod pests, including economically important fruit mite species. The region is important not only for apple production but also as a model for the development of locally adapted and biodiversity-oriented orchard protection systems [70,71]. Because Southeastern Kazakhstan represents both a major commercial apple-producing region and part of the center of origin of domesticated apple, sustainable pest management strategies developed under its agroecological conditions may provide valuable guidance for apple production systems throughout Central Asia [70,71].

Apple production occupies a prominent position within Kazakhstan’s horticultural sector. The southern and southeastern regions of the country, particularly Almaty and Zhetysu, provide the most favorable climatic conditions for apple cultivation and contain a substantial proportion of Kazakhstan’s commercial orchards. Kazakhstan has approximately 35.7 thousand hectares of apple plantations, with apple production concentrated largely in the southeastern foothill regions associated with the center of origin of *Malus sieversii*, the primary progenitor of the cultivated apple [73,74,75,76]. According to national agricultural statistics, these regions account for the majority of the country’s apple production and include both intensive commercial orchards and traditional production systems. The most important arthropod pests affecting apple orchards include codling moth (*Cydia pomonella*), aphids, leafrollers, and phytophagous mites. Among mite pests, species belonging to the genera *Panonychus*, *Tetranychus*, and eriophyid mites have been reported in regional orchards and are considered important constraints to sustainable apple production. Despite their economic significance, comprehensive information on species distribution, population dynamics, resistance development, and biological control potential under local conditions remains limited, emphasizing the need for region-specific research and long-term monitoring programs. Although several recent studies have investigated beneficial arthropods in apple orchards of southeastern Kazakhstan, they have primarily focused on natural enemies of lepidopteran pests rather than on predator–prey interactions involving phytophagous mites, indicating that the biological control of fruit mites under local orchard conditions remains insufficiently studied [77].

In recent decades, the modernization and intensification of orchard production systems have altered pest dynamics throughout the region. Increased orchard density, expansion of commercial production, and changing climatic conditions have created new challenges for sustainable pest management. Among these challenges, fruit mite infestations have become increasingly important because of their potential to reduce photosynthetic activity, impair fruit quality, and increase production costs.

### 8.1. Apple Production Systems and Regional Characteristics

Apple production in Southeastern Kazakhstan is concentrated primarily in irrigated orchard systems located within foothill and mountain-valley agroecosystems. These orchards experience significant seasonal temperature fluctuations, relatively low precipitation during the growing season, and increasing frequency of drought periods [71].

Such conditions may favor the development of phytophagous mites, particularly during warm and dry summers. Similar patterns have been reported in other temperate fruit-growing regions where elevated temperatures accelerate mite development, increase reproductive rates, and contribute to more frequent outbreaks [25]. At the same time, orchard systems in Kazakhstan remain highly heterogeneous. Commercial orchards often coexist with smallholder farms and semi-intensive production systems, resulting in considerable variation in pest management practices [75,76]. This heterogeneity creates both challenges and opportunities for implementing regionally adapted Integrated Pest Management (IPM) programs. Recent studies on apple rootstocks grown under Kazakhstani field conditions further demonstrate the diversity of orchard production systems and the importance of developing locally adapted management practices [78].

### 8.2. Fruit Mite Species and Current Management Practices

Published surveys from southeastern Kazakhstan have documented the occurrence of *Panonychus ulmi*, *Tetranychus urticae*, and eriophyid mites in commercial apple orchards, although comprehensive information on their distribution and ecology remains limited [6,9,20].

Current management practices remain predominantly dependent on chemical control. In many orchards, pesticide applications continue to serve as the primary response to visible pest infestations. While such approaches may provide short-term suppression, they often fail to address underlying ecological drivers of pest outbreaks. At the same time, research on Kazakhstani apple cultivars has demonstrated increasing scientific interest in the characterization of local germplasm and resistance-associated traits, providing an important foundation for the development of regionally adapted orchard protection strategies [78].

A critical challenge is the limited availability of long-term regional datasets concerning mite population dynamics, predator–prey interactions, and resistance development. Consequently, management decisions are frequently based on generalized recommendations developed under different agroecological conditions rather than locally validated evidence. This situation highlights an important knowledge gap. Sustainable management strategies developed for Western Europe or North America cannot necessarily be transferred directly to Central Asian production systems without regional adaptation.

### 8.3. Knowledge Gaps in Central Asian Fruit Mite Management

Despite the economic importance of apple production in Central Asia, relatively few published studies have investigated fruit mite biodiversity, population dynamics, biological control, and acaricide resistance under regional conditions [79]. Among the studies included in this review, only a limited number focused on Central Asian apple production systems, and none reported long-term resistance surveillance or comprehensive monitoring programs for fruit mites [80]. In contrast, Europe, North America, and China have developed well-established IPM frameworks supported by long-term pest monitoring and resistance surveillance programs, providing a strong scientific basis for sustainable fruit mite management [81,82].

Consequently, the distribution, seasonal population dynamics, and economic importance of key fruit mite species in Central Asian apple orchards remain insufficiently documented [79,83]. Another major limitation is the lack of locally validated economic thresholds for fruit mite management. Although regional monitoring programs have been developed for some economically important apple pests in southeastern Kazakhstan, comparable threshold-based decision systems for phytophagous mites remain unavailable [84]. Therefore, the economic thresholds currently applied for fruit mite management are largely extrapolated from studies conducted in Europe and North America under different climatic, agronomic, and production conditions and may not accurately reflect the ecological characteristics of apple orchards in southeastern Kazakhstan and other parts of Central Asia [80,85]. Consequently, the development and field validation of region-specific economic thresholds should be considered a priority for future research to support evidence-based IPM implementation in the region [80,85].

Similarly, information on predator–prey interactions, phytoseiid mite diversity, and the effectiveness of conservation biological control under local orchard conditions remains scarce [79,86,87]. Resistance monitoring represents another major knowledge gap. Although acaricide resistance in *Panonychus ulmi* and *Tetranychus urticae* has been extensively documented worldwide, systematic surveillance programs and molecular resistance monitoring have not yet been widely implemented in Central Asia [24,39,79,88]. Consequently, resistance may remain undetected until substantial declines in field efficacy occur, increasing the risk of control failures and further selection for resistant populations [32,39,88].

Addressing these knowledge gaps should be considered a priority for future research. Particular attention should be directed toward long-term monitoring of mite populations, regional resistance surveillance, biodiversity assessments of natural enemies, and the development of locally validated economic thresholds to support sustainable IPM implementation under Central Asian conditions [89,90,91].

A further limitation of the present review is the limited availability of primary field-based studies from Kazakhstan and other Central Asian countries. Most regional publications provide general, taxonomic, or indirect information, whereas long-term investigations of fruit mite population dynamics, acaricide resistance monitoring, molecular resistance diagnostics, and natural enemy diversity remain scarce. Consequently, the regional conclusions presented in this review should be interpreted as a synthesis of the currently available evidence rather than a comprehensive representation of local fruit mite ecology and management. Future research should therefore prioritize coordinated field surveys, long-term population monitoring, standardized resistance surveillance, molecular diagnostics, and systematic assessments of phytoseiid mites and other natural enemies across the major apple-growing regions of Kazakhstan and Central Asia.

### 8.4. Climatic Change and Emerging Pest Risks

Climate change is expected to significantly influence future fruit mite management in southeastern Kazakhstan, where apple production is closely associated with the unique ecosystems of *Malus sieversii* and is increasingly exposed to rising temperatures and greater climatic variability [72,92]. Rising temperatures, altered precipitation patterns, and more frequent drought events may affect both phytophagous mite populations and their natural enemies [89,90]. Several studies have demonstrated that warming conditions can accelerate the development and increase the number of annual generations of spider mites, thereby increasing the risk of more frequent and severe outbreaks [25,70]. Climate change may also modify predator–prey interactions because predatory mites and other beneficial arthropods often respond differently to changing environmental conditions than their prey [89]. Consequently, future IPM strategies for southeastern Kazakhstan should integrate climate-adaptive monitoring, ecological forecasting, and conservation biological control to improve orchard resilience under changing environmental conditions [90,91].

### 8.5. Opportunities for Sustainable IPM Implementation

The transition toward sustainable fruit mite management in Kazakhstan offers considerable opportunities. Several factors favor the development of ecologically based IPM systems: relatively high orchard biodiversity in some production areas; increasing awareness of sustainable agriculture; expanding research capacity in plant protection and agroecology; growing interest in reducing pesticide dependence [76].

Biological control represents a particularly promising direction. Predatory mites already occur naturally within many orchard ecosystems, suggesting that conservation biological control may provide a cost-effective strategy for enhancing pest suppression [12,46].

Habitat management, cover cropping, and selective pesticide use could further strengthen ecological regulation mechanisms while reducing selection pressure for resistance development. Moreover, integration of monitoring programs and economic thresholds would improve decision-making and reduce unnecessary pesticide applications. A major advantage for Kazakhstan is that many commercial orchards are still undergoing technological modernization and sectoral development, creating opportunities to incorporate IPM principles during system development rather than attempting to retrofit highly pesticide-dependent production systems [75,76].

### 8.6. Research Needs and Future Perspectives

Despite increasing attention to sustainable agriculture, several research gaps remain. First, comprehensive surveys of fruit mite diversity and distribution are required to establish reliable baseline information for regional pest management. Second, studies examining predator–prey interactions under local environmental conditions are needed to evaluate the potential of biological control agents. Third, resistance monitoring programs remain largely underdeveloped. Considering the global increase in acaricide resistance among phytophagous mites, early implementation of resistance surveillance would provide substantial long-term benefits. In addition, further research on local apple germplasm and resistance-associated traits may support the development of cultivars better adapted to regional biotic stresses and sustainable orchard management programs. Molecular characterization of Kazakhstani apple cultivars has already provided valuable information for breeding and resistance-oriented research [78]. Recent assessments of fire blight introduction into wild apple forests of Kazakhstan further highlight the importance of conserving native apple genetic resources and strengthening region-specific phytosanitary and orchard management strategies [93,94]. Finally, future research should move beyond evaluating individual control methods and instead focus on developing integrated orchard protection systems. Such systems should combine biological control, resistance management, habitat diversification, economic threshold-based decision making, and climate adaptation strategies.

#### Critical Perspective

A recurring limitation of pest management programs in many developing and transition economies is the tendency to adopt technologies developed elsewhere without sufficient regional validation. In the context of Southeastern Kazakhstan, the most significant challenge is therefore not the absence of available control tools but rather the lack of locally generated ecological knowledge necessary for their optimal integration.

Consequently, the future of fruit mite management in Southeastern Kazakhstan should focus on the development of region-specific IPM systems that reflect local climatic conditions, orchard structures, biodiversity resources, and socioeconomic realities. Such an approach would contribute not only to sustainable pest suppression but also to the long-term resilience and competitiveness of Kazakhstan’s apple industry.

## 9. Emerging Trends and Future Perspectives

The management of fruit mites in apple orchards is undergoing a significant transition from pesticide-dependent control toward knowledge-intensive, ecologically based pest management systems. Advances in ecology, molecular biology, precision agriculture, and digital technologies are creating new opportunities to improve fruit mite monitoring, resistance management, and decision-making, thereby enhancing the sustainability and resilience of apple orchard protection [89,90,91].

Future fruit mite management is expected to rely increasingly on integrated systems that combine biological regulation, resistance prevention, ecological engineering, and digital monitoring technologies. Such approaches are particularly important in the context of climate change, increasing pesticide restrictions, and growing demand for environmentally sustainable fruit production [25,34,63].

### 9.1. Precision Agriculture and Digital Monitoring

One of the most promising developments in modern fruit mite management is the application of precision agriculture technologies to improve the early detection and monitoring of phytophagous mite infestations in apple orchards. Conventional monitoring of *Panonychus ulmi* and *Tetranychus urticae* relies primarily on repeated visual inspection of leaves, which is labor-intensive, time-consuming, and may fail to detect localized infestations during the early stages of population development [95].

Recent advances in remote sensing, unmanned aerial vehicles (UAVs), wireless sensor networks, and digital decision-support systems provide new opportunities for monitoring orchard conditions and detecting plant stress associated with mite feeding before visible symptoms become widespread [95]. High-resolution multispectral and hyperspectral imaging, together with machine-learning algorithms and artificial intelligence (AI), have demonstrated considerable potential for identifying subtle changes in leaf reflectance, chlorophyll content, canopy temperature, and photosynthetic activity that may indicate early spider mite infestations or mite-induced plant stress [96,97].

Although most digital monitoring technologies have been developed primarily for plant diseases and insect pests, these approaches are increasingly being adapted for the detection of arthropod pests, including phytophagous mites, and may substantially improve the precision and timeliness of Integrated Pest Management (IPM) decision-making [95,96,97]. Earlier detection of localized mite outbreaks could facilitate more targeted acaricide applications, reduce unnecessary pesticide use, improve the conservation of natural enemies, and ultimately contribute to more sustainable fruit mite management [81,91].

Nevertheless, the implementation of precision agriculture technologies in commercial apple orchards remains limited by high equipment costs, technical complexity, insufficient validation under diverse orchard conditions, and limited availability of region-specific decision-support models. Future research should therefore focus on validating digital monitoring tools specifically for fruit mite management, integrating remote sensing with resistance surveillance and biological control programs, and developing affordable technologies suitable for both commercial and smallholder orchards [90,91,95].

### 9.2. Climate Change and Adaptive Pest Management

Future fruit mite management will increasingly depend on the development of adaptive Integrated Pest Management (IPM) strategies capable of responding to climate-driven changes in orchard ecosystems. Rather than relying on fixed management schedules, adaptive IPM incorporates climate-informed monitoring, predictive forecasting, and flexible decision-making to optimize intervention timing under changing environmental conditions [90].

Climate change is expected to influence not only the phenology and population dynamics of phytophagous mites but also the performance of biological control agents. Predatory mites and their prey may respond differently to increasing temperatures, altered precipitation regimes, and more frequent climatic extremes, potentially modifying predator–prey interactions and the long-term effectiveness of conservation biological control [89,90]. Consequently, future IPM programs should integrate climatic forecasting models, long-term ecological monitoring, and region-specific decision-support systems to improve the resilience of apple orchard protection. Such adaptive approaches will help optimize the timing of monitoring and control measures, reduce unnecessary pesticide applications, and maintain sustainable fruit mite management under increasingly variable environmental conditions [90,91].

### 9.3. Future Perspectives for Sustainable Fruit Mite Management

Future advances in fruit mite management are expected to increasingly integrate ecological, technological, and molecular approaches within adaptive Integrated Pest Management (IPM) frameworks. Conservation biological control, habitat diversification, and the enhancement of predatory mite populations are likely to play a greater role in reducing dependence on acaricides while improving the resilience of orchard ecosystems [12,89].

At the same time, molecular diagnostics and resistance surveillance tools offer new opportunities for the early detection of resistance-associated mutations, allowing growers to adjust management strategies before widespread control failures occur [39,80]. However, these technologies should complement rather than replace ecological approaches, as sustainable resistance management ultimately depends on reducing selection pressure through integrated control strategies [32,43].

Future IPM programs should increasingly integrate biological control, resistance management, precision agriculture, and digital technologies within a unified framework tailored to regional orchard conditions. Such integrated approaches are expected to enhance the long-term sustainability of apple production while conserving biodiversity and improving the effectiveness of fruit mite management [89,90,91].

#### Critical Perspective

A recurring theme throughout the evolution of pest management has been the expectation that new technologies will provide definitive solutions to pest problems. Historical experience demonstrates that such expectations are rarely fulfilled. Whether chemical pesticides, biological agents, or digital technologies, individual innovations tend to lose effectiveness when implemented in isolation.

The most promising future direction is therefore not the development of a single transformative technology but the integration of multiple complementary approaches into resilient and adaptive orchard protection systems. Such systems are expected to provide more durable, environmentally sustainable, and economically viable solutions for fruit mite management than any individual control strategy alone.

Consequently, future research should prioritize interdisciplinary approaches that combine ecological knowledge, technological innovation, and region-specific adaptation. These efforts will be essential for ensuring sustainable apple production under increasingly complex environmental and agricultural conditions.

### 9.4. Research Priorities for Central Asia

Although substantial progress has been achieved in fruit mite management worldwide, important knowledge gaps remain in Central Asian apple-producing regions. Future research efforts should focus on developing regionally adapted pest management strategies that reflect local agroecological conditions, climatic variability, and orchard management practices.

Several research priorities can be identified for the sustainable management of fruit mites in Central Asia:

1. Resistance monitoring programs: Long-term surveillance of acaricide susceptibility in *Panonychus ulmi*, *Tetranychus urticae*, and eriophyid mite populations is needed to enable early detection of resistance development and support evidence-based resistance management.
2. Development of local economic thresholds: Existing threshold values are largely derived from studies conducted in Europe and North America. Region-specific thresholds should be established under Central Asian climatic and production conditions to improve decision-making and reduce unnecessary pesticide applications.
3. Biodiversity and natural enemy surveys: Comprehensive assessments of predatory mites and other beneficial arthropods are required to better understand their ecological roles and potential contributions to biological control in regional orchard ecosystems.
4. Climate-adaptive pest management: Future studies should investigate how increasing temperatures, altered precipitation patterns, and climatic variability influence fruit mite population dynamics, biological control efficiency, and resistance development.
5. Digital monitoring and precision agriculture: The integration of remote sensing, digital scouting tools, wireless sensor networks, and predictive models may improve early detection of pest outbreaks and support precision IPM implementation.
6. Conservation biological control and habitat management: Research should evaluate the effectiveness of cover crops, flowering strips, refuge habitats, and ecological engineering strategies for enhancing natural enemy populations and strengthening biological regulation mechanisms.

Addressing these priorities will contribute to the development of resilient, environmentally sustainable, and region-specific Integrated Pest Management programs capable of supporting long-term apple production in Central Asia under changing agricultural and climatic conditions. Collectively, these priorities provide a roadmap for the development of regionally adapted, climate-resilient, and ecologically sustainable fruit mite management programs in Central Asia.

## 10. Conclusions

Fruit mites remain among the most economically important arthropod pests in apple orchards due to their rapid population growth, high reproductive potential, and strong capacity to develop resistance to acaricides. Based on the literature analyzed in this review, several key conclusions can be drawn. First, biological control should be regarded as the ecological foundation of sustainable fruit mite management. Predatory mites, conservation biological control, and habitat diversification provide effective opportunities for suppressing pest populations while reducing pesticide dependence. Second, Integrated Pest Management (IPM) represents the most effective framework for long-term orchard protection. Successful IPM programs combine monitoring, economic thresholds, biological control, cultural practices, selective pesticide use, and resistance management into a coordinated decision-making system. Third, chemical control remains an important component of fruit mite management; however, its role should shift from a primary control strategy to a targeted supporting tool within broader IPM programs. Selective acaricides applied according to monitoring-based recommendations can contribute to effective pest suppression while minimizing impacts on natural enemies. Fourth, acaricide resistance has become a global challenge threatening the sustainability of fruit mite management. Long-term resistance mitigation requires reduced selection pressure, mode-of-action diversification, conservation of biological control agents, and continuous resistance monitoring. Fifth, Southeastern Kazakhstan represents a strategically important but insufficiently studied region for fruit mite management. The development of locally adapted ecological and IPM-based protection systems should be considered a priority for improving the sustainability and competitiveness of regional apple production. Finally, future research should focus on integrating ecological knowledge, biodiversity conservation, climate adaptation, precision agriculture, digital monitoring technologies, and resistance management into resilient orchard protection systems. Sustainable fruit mite management will ultimately depend on the successful integration of biological control, resistance management, ecological engineering, and monitoring-based decision-making within resilient orchard ecosystems.

## Figures and Tables

**Table 1:** Major fruit mite species affecting apple orchards and their direct economic significance.

Species	Origin	Distribution	Direct Economic Impact	Key Biological Traits	Major Management Challenges
*Panonychus ulmi*	Europe	Temperate apple-growing regions worldwide	Reduced photosynthetic activity, leaf bronzing, and yield loss	Multiple generations, high reproductive rate	Rapid resistance development
*Tetranychus urticae*	Presumed Eurasian origin	Cosmopolitan	Leaf bronzing, defoliation, and fruit quality reduction	Polyphagous, rapid reproduction, multiple generations	Severe acaricide resistance development, broad host range
*Aculus schlechtendali*	Europe	Europe, Asia, and North America	Russeting and fruit quality reduction	Small size, concealed feeding habit	Difficult monitoring and threshold determination

Note: The relative economic importance of each species was assessed according to the reported frequency of outbreaks, potential yield losses, geographical distribution, and management challenges documented in the reviewed literature.

**Table 2:** Major acaricide groups used in Integrated Pest Management (IPM) of fruit mites in apple orchards: modes of action, target stages, major limitations, and resistance risk.

Acaricide Group	Representative Active Ingredients	Mode of Action	Target Stage	Major Limitations	Resistance Risk
METI acaricides	Fenpyroximate, Pyridaben	Inhibition of mitochondrial electron transport	Mobile stages	Limited ovicidal activity; negative effects on predatory mites	High
Lipid synthesis inhibitors	Spiromesifen, Spirodiclofen	Lipid biosynthesis inhibition	Eggs, larvae, nymphs	Slow action; limited efficacy against adults	Moderate
Growth regulators	Hexythiazox, Etoxazole	Inhibition of molting and development	Eggs and immature stages	Ineffective against adults	Moderate
Avermectins	Abamectin	Activation of glutamate-gated chloride channels	Multiple mobile stages of mites	Short residual activity; toxicity to beneficial arthropods	High
Pyrethroids	Various compounds	Sodium channel modulators	Broad-spectrum arthropods	Disruption of biological control; secondary pest outbreaks	High
Complex II electron transport inhibitors (IRAC Group 25)	Cyflumetofen	Inhibition of mitochondrial complex II electron transport	Mobile stages	Resistance may develop under repeated use; limited ovicidal activity	Moderate
Mitochondrial ATP synthase inhibitors	Cyenopyrafen	Inhibition of ATP synthesis	Mobile stages	Limited ovicidal activity; resistance management required	Moderate

Note: Resistance risk was classified qualitatively according to the number of documented resistance cases, reports of cross-resistance, known resistance mechanisms, and current IRAC resistance management recommendations described in the reviewed literature. The categories High and Moderate represent qualitative assessments based on the available published evidence rather than quantitative thresholds.

**Table 3:** Reported acaricide resistance in major fruit mite species affecting apple orchards.

Species	Acaricide Group	Example Active Ingredients	Reported Resistance Mechanisms	Regions with Documented Resistance
*Panonychus ulmi*	METI acaricides	Fenpyroximate, Pyridaben	Target-site mutations, enhanced detoxification enzymes	Europe, North America, China
*Panonychus ulmi*	Growth regulators	Hexythiazox	Reduced susceptibility, metabolic resistance	Europe, Asia
*Tetranychus urticae*	Avermectins	Abamectin	CYP450-mediated detoxification, target modification	Worldwide
*Tetranychus urticae*	Lipid synthesis inhibitors	Spiromesifen, Spirodiclofen	Metabolic resistance	Europe, China, Turkey
*Tetranychus urticae*	Pyrethroids	Bifenthrin and others	Knockdown resistance and detoxification mechanisms	Worldwide

**Table 4:** Principal Components of Integrated Pest Management (IPM) for Fruit Mites in Apple Orchards.

PM Component	Primary Role in Fruit Mite Management	Main Advantages	Main Limitations	Contribution to Sustainable Management
Monitoring and economic thresholds	Early detection of *Panonychus ulmi* and *Tetranychus urticae* outbreaks and optimization of intervention timing	Reduces unnecessary acaricide applications; supports decision-making	Requires regular monitoring and technical expertise	Very High
Selective acaricide use	Rapid suppression of mite populations exceeding economic thresholds	High efficacy; compatible with IPM when selective products are used	Resistance development; possible non-target effects on natural enemies	Moderate
Biological control (including conservation biological control)	Suppression of phytophagous mites through predatory mites, *Stethorus* spp., and other natural enemies	Environmentally friendly; preserves biodiversity; reduces pesticide dependence	Performance influenced by climate, pesticide selectivity, and habitat conditions	High
Cultural practices	Reduce pest establishment and improve orchard resilience through pruning, balanced fertilization, irrigation management, and sanitation	Preventive; compatible with all other IPM tactics	Variable effectiveness depending on orchard conditions	High
Resistance management	Delay evolution of acaricide resistance through mode-of-action rotation, monitoring, and reduction in selection pressure	Extends acaricide longevity; improves long-term control sustainability	Requires coordinated planning and continuous monitoring	Very High

## Data Availability

No new datasets were generated or analyzed during the current study. All information presented in this review was obtained from publicly available scientific literature cited in the reference list.

## Abbreviations

The following abbreviations are used in this manuscript:

AI	Artificial Intelligence
IPM	Integrated Pest Management
IRAC	Insecticide Resistance Action Committee
METI	Mitochondrial Electron Transport Inhibitor
UAV	Unmanned Aerial Vehicle
MOA	Mode of Action
PCR	Polymerase Chain Reaction
DNA	Deoxyribonucleic Acid
GIS	Geographic Information System
FAO	Food and Agriculture Organization of the United Nations
UAVs	Unmanned Aerial Vehicles
